# Regional Differences in Biceps Femoris Long Head Stiffness during Isometric Knee Flexion

**DOI:** 10.3390/jfmk6010018

**Published:** 2021-02-10

**Authors:** João R. Vaz, Tiago Neto, José Pedro Correia, Jorge Infante, Sandro R. Freitas

**Affiliations:** 1 Neuromuscular Research Lab, Faculty of Human Kinetics, University of Lisbon, 1499-002 Lisbon, Portugal; josepedro87@hotmail.com (J.P.C.); sfreitas@fmh.ulisboa.pt (S.R.F.); 2 CIPER, Faculty of Human Kinetics, 1499-002 Lisbon, Portugal; jinfante@fmh.ulisboa.pt; 3 Department of Physiotherapy, LUNEX International University of Health, Exercise and Sports, 4671 Differdange, Luxembourg; tiago.neto@lunex-university.net

**Keywords:** shear wave elastography, shear modulus, muscle stiffness, hamstrings, regionalization

## Abstract

This study sought to investigate whether the stiffness of the biceps femoris long head differs between proximal and distal regions during isometric knee flexion at different contraction intensities and muscle lengths. Twelve healthy individuals performed knee flexion isometric contractions at 20% and 60% of maximum voluntary isometric contraction, with the knee flexed at 15 and 45 degrees. Muscle stiffness assessment was performed using ultrasound-based shear wave elastography. Proximal and distal regions of the biceps femoris long head were assessed. Biceps femoris long head muscle showed a greater stiffness (i) in the distal region, (ii) at higher contraction intensity, and (iii) at longer muscle length. The proximal-to-distal stiffness ratio was significantly lower than 1 (i.e., heterogenous) at lower contraction intensity regardless of the muscle length. However, this was not observed at higher contraction intensity. This study is the first to show heterogeneity in the active stiffness of the biceps femoris long head. Given the greater incidence of injury at the proximal region of biceps femoris long head, this study opens new directions for future research. Additionally, the present study results indicate that studies assessing muscle stiffness at one single muscle region should be interpreted with caution.

## 1. Introduction

Skeletal muscle physiological properties have recently been reported to be heterogenous along its length in an active condition. Particularly, recent studies have revealed neural and metabolic differences between regions within the biceps femoris long head (BFlh) [1,2,3,4,5,6]. By using electromyography, a different activity was observed between the proximal and distal BFlh regions during common hamstring exercises [4,5]. In addition, a heterogeneous T2 relaxation response (assessed using functional magnetic resonance imaging) along BFlh muscle length was observed after a bout of exercise [2,7]. Together, these recent findings suggest that the central nervous system may independently control different regions of the BFlh. Moreover, it has been reported that BFlh is innervated by more than one motor nerve branch [1]. Regardless, the activation of different regions appears to be task-dependent (e.g., hip extension vs. knee flexion). However, it is unclear whether the observed heterogeneous response translates into different regional mechanical properties, specifically in terms of active muscle stiffness.

Through the use of ultrasound-based shear wave elastography (SWE), several studies have recently investigated the stiffness of different muscles in vivo during contraction (i.e., active stiffness) through estimates of localized shear modulus [8,9,10]. Although the validity of these measurements has not been fully explored, the aforementioned studies have only assessed the muscle stiffness at a single region, assuming stiffness homogeneity throughout the muscle length. The assumption of a homogenous muscle stiffness response throughout its length was accepted for several years [11]. However, some studies have shown that it is not the case [12,13]. For example, Sant et al. [13] have shown a greater muscle stiffness in the distal region of the human medial gastrocnemius muscle during passive ankle dorsiflexion, and such difference was particularly evident with a greater muscle stretch. In other words, muscle length appears to affect muscle stiffness during passive motion. This was also previously observed in the tibialis anterior muscle during contraction [14]. Sasaki et al. [14] showed that muscle stiffness increased with fascicle-length increase. Similarly, these authors showed a linear relationship between force and muscle stiffness, suggesting that muscle stiffness increased with contraction intensity. On the other hand, Freitas et al. [15] showed no regional differences in passive muscle stiffness in the quadriceps muscles during passive knee flexion. However, it remains to be determined whether human BFlh active stiffness is region-dependent during contraction and how muscle length and contraction intensity may affect it.

This study aimed to investigate whether BFlh stiffness is region-dependent during knee flexion isometric contractions with different contraction intensities and muscle lengths. Considering the aforementioned previous findings, we tested the hypothesis that BFlh would exhibit a greater active stiffness (i) at the distal region, (ii) with higher contraction intensity, and (iii) at a longer muscle length, regardless of the muscle region assessed. Additionally, we explored how the BFlh proximal-to-distal active ratio would differ, and how it would be affected by contraction intensity and muscle length. We hypothesized this ratio would be closer to 1 at greater contraction intensity and at shorter muscle length, i.e., more homogenous.

## 2. Materials and Methods

### 2.1. Participants

Twelve physically active male individuals participated in this study (25 ± 2 yrs.; 1.68 ± 0.08 m; 66.8 ± 11.9 kg). Participants reported no previous history of hamstring strain injury nor any other lower limb musculoskeletal injury within the previous 6 months. Signed informed consent was obtained from each participant. This study was approved by the local Ethics Committee (n°21, 25 October 2016), in accordance with the Declaration of Helsinki.

### 2.2. Instrumentation

Knee joint torque in one randomly chosen lower limb was measured at 1 kHz using an isokinetic dynamometer (Biodex System 3, Shirley, NY, USA). Participants were tested in prone position with the hip neutral (i.e., 0°) and the knee flexed at two angles: 15° and 45°. The ankle of the tested limb was immobilized in neutral position (i.e., 90°) with a thermoplastic cast and fixed using tape. The lateral femoral condyle was aligned with the dynamometer axis, and the dynamometer pad was fixed to the distal leg. The contralateral lower limb rested with the joints in a neutral position during the testing.

The shear modulus of the BFlh was assessed using an ultrasound scanner (Aixplorer, v11; Supersonic Imagine, Aix-en-Provence, France) in SWE mode (musculoskeletal preset, penetrate mode, smoothing level 5, persistence off; scale: 0–800 kPa), coupled with a linear transducer array (4–15 MHz. Super Linear 15-4, Vermon, Tours, France). The SWE technique has been previously described in detail [16]. The ultrasound transducer was placed at ≈25% and ≈75% of the proximal-to-distal BFlh length. BFlh length was determined as the distance between proximal and distal muscle–tendon junctions, as assessed through B-mode ultrasound imaging. Care was taken to align the transducer with the muscle fascicle orientation during contraction. To ensure a stable and consistent muscle shear modulus measurement during across repeated trials, a plastic cast designed for the transducer was fixed to the skin superficial to each muscle region. The transducer was manually held by an examiner during the measurements, with minimal pressure against the skin. The shear modulus of each muscle region was measured during a 15-s submaximal isometric contraction. Before instructing the participant to contract, 10 s of shear modulus were collected to determine resting values. Both B-mode and elastogram image sequences were recorded during the tests.

### 2.3. Protocol

At participants’ arrival, the custom-made casts were placed over the muscle regions of interest. A set of ten submaximal contractions was performed as warm-up and for familiarization with the equipment and contraction intensity. Five minutes before data collection, two knee flexion maximum voluntary isometric contraction (MVIC) trials of approximately 3-s duration were performed with the knee at 15°. This was required to determine the torque corresponding to the contraction intensity under investigation. A minimum of 1-min resting period was given between trials. Then, the participants performed sixteen knee flexion isometric contractions, i.e., 2 trials × 2 contraction intensities (20% and 60%) × 2 knee angles (15° or 45°) × 2 muscle regions. Visual feedback was given to the participants regarding the joint torque attained. The order of the trials was randomized. A minimum of 1 min of rest was given between trials. At the end of the protocol, two MVIC trials of 3-s duration were performed to determine whether the participants fatigued as assessed by a decrease in peak torque at 15° of knee flexion.

### 2.4. Data Collection and Processing

Data were synchronized using an external switch that triggered data capture from all equipment simultaneously and acquired using a Biopac acquisition system (MP100, Santa Barbara, CA, USA). All further data processing was performed using Matlab^®^ (Natick, MA, USA). For the shear modulus calculation of each muscular region, the video clips exported from the ultrasound software were sequenced into .jpeg images. Each pixel of the color map was converted into a value of the elastic modulus based on the recorded color scale, using image processing. The largest region of interest in the elastogram window was determined by avoiding aponeurosis and tissues artefacts, and the shear modulus values were averaged for a representative muscle value. The average muscle elastic modulus was divided by 3 to estimate the muscle shear elastic modulus [16]. Note that in the present document, passive and active muscle stiffness terminology are used to refer to shear elastic modulus at rest and during contraction, respectively. For each trial, the shear modulus values observed during a stable 10-s interval of the 15-s isometric contraction were averaged and used for statistical analysis. The proximal-to-distal BFlh ratio was calculated for passive muscle stiffness (PD_PASSIVE_) and active muscle stiffness (PD_ACTIVE_) variables and used for analysis. Note that a greater proximal-to-distal ratio reflects a higher value in the proximal region relative to the distal.

In addition to all the studied parameters, we have also determined the probe–fascicle angle to allow a proper interpretation of our results. For this, we have measured such an angle for three different fascicles within the same picture and averaged it. We have conducted this analysis for all files. This was measured in two independent conditions: at rest and during contraction.

### 2.5. Statistical Analysis

Data analysis was performed using IBM SPSS Statistics 22.0 (IBM Corporation, New York, NY, USA). Normality was confirmed using the Shapiro–Wilk test. The repeatability of the BFlh shear modulus assessment was determined by calculating the intraclass coefficient correlation (ICC2,1) and the standard error of measurement (SEM) [17]. To examine whether the protocol induced knee flexor fatigue, MVICs before and after the protocol were compared using a paired *t*-test. To determine whether a regional BFlh stiffness existed regardless of contraction intensity and joint position, a three-way repeated measures ANOVA [region (proximal, distal) × intensity (20%, 60%) × position (knee flexion: 15°, 45°)] was performed. Additionally, to determine whether proximal-to-distal BFlh ratios varied depending on contraction intensity and joint position, a two-way repeated measures ANOVA (intensity (20%, 60%) × joint position (15°, 45°)) was performed for passive and active muscle stiffness. In case of a significant main effect or interaction, Bonferroni post hoc tests were performed. One sample Student’s *t*-tests with one as a reference value was also performed to investigate if PD_PASSIVE_ and PD_ACTIVE_ were homogenous or heterogenous. Homogeneity was considered when not different from 1.0 (i.e., *p* > 0.05). To test whether the probe–fascicle angle was different between regions, we used a paired sample’s *t* test. Additionally, we have calculated the Cohen’s D as an effect size measure. Significance for all statistics was set at *p* < 0.05.

## 3. Results

**Figure 1:**
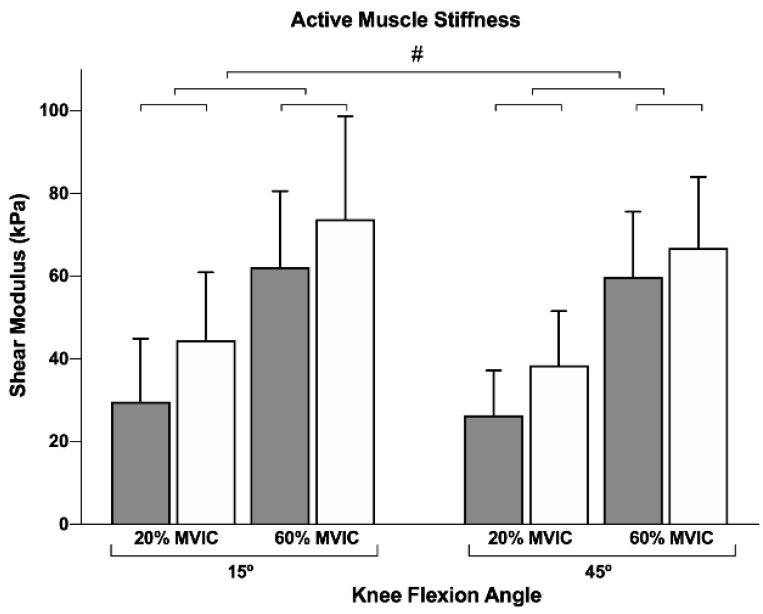
Biceps femoris long head active stiffness at proximal (grey bars) and distal (white bars) regions during knee flexion contractions at 15° and 45° of knee flexion and 20% and 60% of MVIC_15°_. MVIC_15_—maximum voluntary isometric contraction at 15° of knee flexion. All data are presented as mean ± standard deviation. # indicates a statistically significant difference (*p* < 0.05).

A negligible and non-significant reduction in knee flexor MVIC was observed (−1.2 ± 9.7%, *p* = 0.515), indicating that our results are unlikely to be affected by potential fatigue effects. Additionally, a high to very high repeatability was observed for active stiffness (active stiffness: ICC = 0.89 (0.81–0.94), SEM = 7.7 kPa) and distal (active stiffness: ICC = 0.77 (0.61–0.87), SEM = 11.5 kPa) muscle regions. The active muscle stiffness at both proximal and distal regions can be observed in Figure 1.

### 3.1. Probe–Fascicle Angle

For the at-rest condition, we have observed a greater probe–fascicle angle at the distal region (*p* < 0.001, d = 1.30; 16.68 ± 3.80° and 10.65 ± 4.76° for distal and proximal regions, respectively). Likewise, we also observed a greater probe–fascicle angle at the distal region during contraction (*p* < 0.001, d = 2.04; 20.32 ± 5.49° and 8.96 ± 4.18° for distal and proximal regions, respectively). These results indicate that the probe–fascicle angle was different between proximal and distal regions.

### 3.2. Passive Muscle Stiffness

No interactions (*p* = 0.645, η^2^ = 0.020) were found for passive muscle stiffness. Likewise, there were no significant main effects for muscle region (*p* = 0.439, η^2^ = 0.055) nor joint position (*p* = 0.136, η^2^ = 0.190).

### 3.3. Active Muscle Stiffness

No interactions between factors were observed. Conversely, a main effect was found for all the three factors: muscle region (*p* = 0.047, η^2^ = 0.312), intensity (*p* < 0.001, η^2^ = 0.939), and joint position (*p* = 0.025, η^2^ = 0.379). Pairwise comparisons revealed a greater active muscle stiffness: (i) for distal (55.9 ± 4.6 kPa) compared to the proximal region (44.5 ± 3.8 kPa); (ii) at a higher contraction intensity (34.7 ± 3.1 and 65.6 ± 3.9 kPa for 20% and 60%, respectively); and (iii) for a longer muscle length (52.5 ± 3.9 and 47.8 ± 3.0 kPa for 15° and 45° of knee flexion, respectively). Figure 1 and Table 1 present the mean and standard deviations for all conditions.

### 3.4. Proximal-to-Distal Ratio

Regarding the PDPASSIVE, no joint position main effect was (*p* = 0.565, η^2^ = 0.015). In terms of PDACTIVE, no interaction for intensity x joint position was observed (*p* = 0.926, η^2^ = 0.001), no main effects were found for intensity (*p* = 0.055, η^2^ = 0.295) nor joint position (*p* = 0.475, η^2^ = 0.047). Table 1 presents mean and standard deviation values for both intensities and joint positions.

One sample *t*-tests revealed that muscle stiffness was homogenous at rest (Table 1). In terms of active stiffness, heterogeneity was found at 20% of MVC_15_ (*p* = 0.027 and *p* = 0.044, for 15° and 45° of knee flexion), but homogeneity was found at 60% of MVC_15_ (*p* = 0.318 and *p* = 0.445, for 15° and 45° of knee flexion).

## 4. Discussion

**Figure 2:**
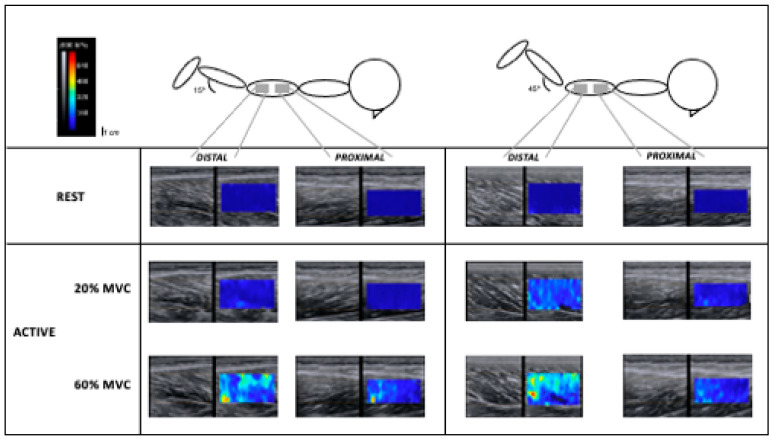
Illustration of one participant’s elastograms under all conditions.

The present study investigated whether BFlh stiffness is region-dependent during isometric knee flexion at different intensities (20% and 60% of MVIC_15°_) and muscle lengths (knee flexed at 15° and 45°). Our initial hypotheses were partially supported. First and foremost, we observed a greater BFlh stiffness at the distal region. Secondly, active muscle stiffness was greater at higher contraction intensity, regardless of the muscle region. Third, BFlh exhibited a lower active stiffness at the shorter muscle length. Lastly, the BFlh active stiffness revealed to be heterogeneous at lower contraction intensity regardless of muscle length (Figure 2 illustrates one participant’s elastrograms).

To the best of our knowledge, this is the first study to investigate human skeletal muscle stiffness in different muscle regions during submaximal isometric contractions. Overall, we found that the distal and proximal regions have different levels of active stiffness, suggesting a heterogeneous stiffness distribution within the BFlh muscle. However, this heterogeneity appears to be intensity-dependent. Importantly, this was not observed in passive muscle stiffness (measured at rest). Instead, we observed homogeneity along BFlh. Therefore, the higher active stiffness observed at the distal region may be explained by its proximity to the joint being mobilized (i.e., knee) and by intramuscular force transmission processes. One possible explanation is that forces generated by the contractile elements of the proximal region are distally transmitted toward the knee joint through non-contractile structures, which likely result in greater forces observed at the distal region [12,18]. Another possibility may be related to BFlh muscle architecture. BFlh architecture is known to vary along its length [19,20]. It is possible that the length changes of BFlh fascicles during contraction are different between muscle regions. However, Bennett et al. [21] have shown a similar relative fascicle shortening between proximal and distal BFlh regions during isometric contractions at different intensities, suggesting that muscle fascicles operate at a similar strain. Another possible explanation refers to the ability of the central nervous system to preferentially activate a certain muscle region. In the present study, the distal region would have been preferentially activated as it was closer to the joint at which the torque was generated. We have found heterogeneity in active stiffness during the lower contraction intensity, suggesting that higher levels of joint torque require a more global actuation of this muscle. Thus, it may be possible that the muscle regionalization is more evident at lower contraction intensities.

Another interesting finding in this study was the observation of a distinct response with the knee flexed at 15° compared to 45°. This may be explained by the skeletal muscle architecture and force–length relationship. BFlh is a pennate muscle and therefore is more sensitive to decreases in muscle length in comparison to fusiform muscles. It has been previously reported that the peak activity of the fusiform knee flexor, semitendinosus, occurs at a greater flexion angle than that of the BFlh [22]. Thus, it is possible that BFlh fascicles have a mechanical disadvantageous length during knee flexion that, despite the higher muscle activity, leads to a stiffness decrement. We speculate that knee flexor muscles with a lower pennation angle and less sensitivity to muscle length changes (e.g., semitendinosus) compensate by increasing stiffness to maintain the torque production. Nonetheless, whether a stiffness alteration occurs between synergistic muscles when changing muscle length is beyond the scope of the present study and remains to be explored.

The present results provide important methodological insights regarding the use of SWE to examine the localized muscle stiffness during contraction. Previous studies have only assessed stiffness at a single muscle region [8,9,10,23], assuming a homogeneous distribution along the muscle’s length. Our results show that such an assumption is flawed and that regionalized differences should be taken into consideration, particularly during low intensity contraction. Therefore, the ultrasound probe placement should be described in detail in future studies. This study also provides scope for a better understanding of skeletal muscle hypertrophy as a consequence of training. The muscular tension evoked during contraction has been proposed to be a primary mechanism to trigger skeletal muscle hypertrophy [24]. Similar to the proposed association between regional hypertrophy and localized activity [25,26], we speculate that the magnitude of regional muscle size may relate to the stiffness during contraction. This topic is worthy of future investigation.

The present study has some limitations. First, only male participants took part of this investigation. Secondly, the BFlh neuromechanical assessment was performed on the ascending limb of the force–length curve [27], indicating that BFlh was operating at a shorter muscle length when the knee was at 45°. It is possible that the results would differ with the BFlh set at a longer length (i.e., descending phase of the force–length curve). The investigation of regional differences along the entire torque–angle curve is an important next step is this area of research. Although recent research has indicated that an increase of fascicles’ pennation angle could attenuate shear wave propagation (i.e., shear modulus decrement), this effect was shown to be small [28,29,30]. Importantly, BFlh has been shown to have a smaller fascicles pennation angle proximally [20]. Indeed, we have shown that the BFlh proximal region exhibits a lower shear modulus for the same contraction intensity, suggesting that the results of the present study are robust. Additionally, we have shown that the fascicle angle relative to probe orientation was smaller at the proximal region of BFlh. Moreover, we have also calculated the shear modulus when the muscle was at rest (passive stiffness). Interestingly, we have shown that passive stiffness is homogenous along BFlh’s length, further suggesting that the differences observed results in the active stiffness are directly related to what occurs when the muscle contracts. Altogether, this strengthens our confidence on the present study’s results.

## 5. Conclusions

This study shows that the active stiffness of the biceps femoris long head is heterogeneous along its length during isometric knee flexion, exhibiting higher stiffness at the distal region. This opens new perspectives to further explore regional adaptations to training and disuse. In particular, given the extensive epidemiological data supporting that biceps femoris long head is most commonly injured at its proximal region, deeper understanding of the regional difference amongst this muscle in athletes with and without previous injury would be interesting to explore. Furthermore, the investigation of in vivo regional differences during active conditions should be extended to other muscles, other joints angles, and joints. This would increase the fundamental knowledge of muscle mechanics, which potentially contribute to a better prevention approach.

## Figures and Tables

**Table 1:** Biceps femoris long head passive and active stiffness in both regions (proximal and distal) across the different conditions, and its proximal-to-distal ratio. Note that for passive stiffness, there was no intensity factor, since it was measure during passive, at rest, condition. All data are presented as mean ± standard deviation. # indicates statistically difference from 1 (*p* < 0.05).

	15° of Knee Flexion		45° of Knee Flexion	
	20% MVC_15°_	60% MVC_15°_	20% MVC_15°_	60% MVC_15°_
***Passive Stiffness***				
Proximal	4.16 ± 1.16		3.96 ± 1.11	
Distal	4.52 ± 0.86		4.09 ± 0.76	
***Active Stiffness***				
Proximal	29.64 ± 15.22	62.13 ± 18.42	26.35 ± 10.79	59.72 ± 15.84
Distal	44.45 ± 16.48	73.76 ± 24.87	38.43 ± 13.09	66.82 ± 17.18
***Proximal-to-Distal Ratio***				
Passive	0.96 ± 0.33		1.01 ± 0.37	
Active	0.71 ± 0.39 #	0.90 ± 0.32	0.75 ± 0.38 #	0.93 ± 0.29

## Data Availability

The data presented in this study are available on request from the corresponding author.
